# An enterohaemorrhagic *Escherichia coli* outbreak spread through the environment at an institute for people with intellectual disabilities in Japan in 2005

**DOI:** 10.5365/wpsar.2017.8.4.010

**Published:** 2019-04-29

**Authors:** Masaki Ota, Taro Kamigaki, Satoshi Mimura, Kazutoshi Nakashima, Takashi Ogami

**Affiliations:** a Research Institute of Tuberculosis, Tokyo, Japan.; b Department of Virology, Graduate School of Medicine, Tohoku University, Sendai, Japan.; c Department of Respiratory Medicine, Japan Self Defense Force Central Hospital, Tokyo, Japan.; d Department of Health Science, Faculty of Sports and Health Science, Daito Bunka University, Saitama, Japan.; e Hokubu Health Office, Oita Prefecture, Oita, Japan.

## Abstract

**Objective:**

An enterohaemorrhagic *Escherichia coli* (EHEC) outbreak at an institute with multiple facilities for children and adults with intellectual disabilities was investigated to characterize the cases and identify risk factors for infection.

**Methods:**

A case was defined as a resident, a staff member or a visitor at the institute from 16 May through 30 June 2005 testing positive for type 2 Vero toxin-producing EHEC O157:H7 (confirmed case) or exhibiting bloody diarrhoea for two or more days (probable case). We collected and analysed demographic, clinical, laboratory and individual behaviour data to identify possible risk factors for infection and infection routes.

**Results:**

We recorded 58 confirmed cases, of which 13 were symptomatic. One probable case was also found. The median age of the patients was 37 years (range: 6–59 years). Thirty-six patients (61%) were male. Thirteen patients (93%) had diarrhoea and six (43%) had abdominal pain. Two developed haemolytic-uraemic syndrome but recovered. All the patients were treated with antibiotics and tested negative after treatment. Some residents had problems with personal hygiene. The residents of one of the facilities who cleaned a particular restroom had 18.0 times higher odds of being infected with EHEC (95% confidence interval: 4.0–102.4) than those who did not.

**Discussion:**

The source of the outbreak could not be identified; however, the infection may have spread through environmental sources contaminated with EHEC. We recommend that institutional settings, particularly those that accommodate people with intellectual disabilities, clean restrooms as often as possible to reduce possible infection from contact with infected surfaces.

## Objectives

Enterohaemorrhagic *Escherichia coli* (EHEC) was first reported in 1983 in the United States of America. (*1*) Infection can cause diarrhoea, haemorrhagic colitis and haemolytic-uraemic syndrome (HUS). (*2*) Outbreaks involving EHEC can be spread through infected food, (*2*) water, (*3*) direct contact with infected humans (*4*) or animals (*5*, *6*) or exposure to infected environments. (*7*) In Japan, EHEC is a reportable communicable disease; the largest outbreak to date was associated with consumption of white radish sprouts affecting about 8400 schoolchildren in 1997. (*8*)

On 6 June 2005, a physician informed a local health office of Oita Prefecture in western Japan of two EHEC cases at an institute for adults and children with intellectual disabilities.

The objectives of the study were to characterize the epidemiology of the cases and identify possible risk factors for EHEC infection in this outbreak.

## Methods

A case was defined as a resident, a staff member or a participant in the activities at the institute for at least one day from 16 May through 30 June 2005 who had a stool specimen that tested positive for Vero toxin type 2 (VT2)-producing EHEC O157:H7 (confirmed case) or exhibited bloody diarrhoea for two days or more (probable case), considering the long incubation period (1 to 9 days) of EHEC.

At the time of the outbreak, the institute had 162 long-term and 13 short-term residents, 20 participants of day care and vocational training and 81 staff members (total 276) in three facilities (Facilities A, B and C). Facilities A and B were for children and adults with intellectual disabilities, respectively, and Facility C was for vocational training and residence for adults with mild intellectual disabilities. Some residents of Facility A attended a school outside the facility run by another organization five days a week. Residents of Facility C worked outside the institute, and about 20 persons who lived outside the institute attended vocational training held at Facility C.

To obtain relevant epidemiological and clinical information, the local health office staff and the authors interviewed the staff members at the time of the outbreak and reviewed charts of the residents and participants at the institute using semi-standardized instruments, to determine demographics (e.g. age, sex), symptoms and signs, date of onset and potential exposure history. Affected patients were referred to a local hospital where we also reviewed patient charts. Environmental samples, including surface swabs of doorknobs, water taps and stair and hand rails, were also collected from the institute by the local health office staff and examined at the Oita Prefecture Hygiene and Environment Centre (OPHEC). (*9*) The local health office collected stool samples from residents, vocational training participants and staff of the school for the intellectually disabled. The samples were examined for EHEC at the local health office and OPHEC using the National Institute of Infectious Diseases’ standard method. (*10*) Staff members of the institute collected stool samples from the kitchen staff that were examined at a private laboratory. We randomly selected half of the strains isolated from the confirmed cases which were further analysed with pulsed-field gel electrophoresis (PFGE). (*11*)

We conducted a nested, unmatched case-control study of the residents, vocational training participants and the staff at each facility. For the case-control study, we defined a case as a person with bacteriologically confirmed EHEC infection. All residents at the facilities who tested negative for EHEC and did not exhibit diarrhoea between 1 and 9 June were chosen as controls. Individuals who had diarrhoea but tested negative for EHEC were not included in the case-control study. We conducted interviews with the patients and controls with the assistance of the staff, if necessary, using a structured questionnaire. Potential risk factors were age; sex; daily living skills, including personal hygiene (whether one was able to wash hands, brush teeth or bathe independently); toilet hygiene (whether one was able to defecate independently or one had allotriophagic behaviours); and needing assistance in taking meals; participating in day care or vocational training; and specific restrooms used or cleaned. Stratified analysis by Mantel-Haenszel method was employed to explore and adjust odds ratios if the univariable analysis revealed statistically significant result (*p*-value < 0.05).

Statistical tests were conducted using R software (The R Foundation for Statistical Computing, Vienna, Austria), and a *p*-value of < 0.05 was considered statistically significant.

### Ethics statement

The investigation was conducted in accordance with the Infectious Disease Control Act of 1999 and the Food Safety Act of 1947 of Japan, which grants the prefectural health director the authority to collect epidemiological information and biologic specimens from patients without obtaining formal consent, in the event of an outbreak of certain confirmed or suspected communicable diseases, including EHEC.

## Results

All 276 residents, staff members, and participants of day care and vocational training had stool specimens collected and examined for EHEC. Fifty-nine cases were reported, of which 58 (98%) were confirmed and one was probable (2%). The probable case was a resident of Facility C who had continuous bloody diarrhoea but had been treated with antibiotics before the stool examination and tested negative for EHEC. Overall, 14 (24%) cases were symptomatic. The median age of the cases was 37 years (range: 6–59 years), and 36 (61%) cases were male. Four staff members exhibited non-bloody diarrhoea during the outbreak period; all four tested negative for EHEC and were eventually determined not to be confirmed or probable cases.

Among the staff, there were five cases (6.2%). No staff members were out ill before the first case report on 1 June. No children in the school, except for those who were residents of Facility A, tested positive for EHEC. There were no reports of any diarrhoea among staff members and children at the school, except for those who were residents of Facility A.

The breakdown of bacteriological test results by facility is shown in **Table 1**. When stratified by sex and location, the infection rate was highest in women residents of Facility C (52.6%, 95% confidence interval [CI]: 28.8–75.5%).

**Table 1:** EHEC O157:H7 positivity among staff, residents and day care participants of an institute for people with intellectual disabilities, Japan, 2005

-			Bacteriologically positive		Total
			n	%	
Facility A	Residents	Male	10	25.0	40
		Female	1	8.3	12
	Participants	Male	0	0	5
		Female	0	0	4
	Staff	Male	0	0	5
		Female	2	9.1	22
	**Subtotal**		**13**	**14.8**	**88**
Facility B	Residents	Male	18	37.5	48
		Female	8	36.4	22
	Participants	Male	0	0	2
		Female	0	0	0
	Staff	Male	2	16.7	12
		Female	0	0	22
	**Subtotal**		**28**	**26.4**	**106**
Facility C	Residents	Male	5	17.8	28
		Female	6	50.0	12
	Participants	Male	1	6.7	15
		Female	4	57.1	7
	Staff	Male	0	0	9
		Female	1	9.1	11
	**Subtotal**		**17**	**20.7**	**82**
**Total**			**58**	**21.0**	**276**

**Fig. 1:**
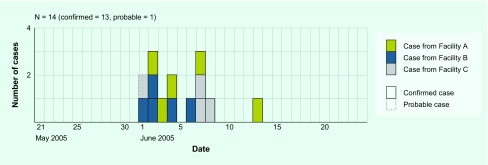
Epidemic curve of symptomatic cases by date of symptom onset during an outbreak of EHEC O157:H7 at an institute for people with intellectual disabilities, Japan, 2005

**Fig. 1** shows the epidemic curve of the 14 symptomatic (13 confirmed and one probable) cases. Four of the five residents who exhibited symptoms and signs from 1 to 2 June participated in a vocational training held on the 1st floor of Facility C; however, we were not able to identify a period of close contact or a possible event that may have transmitted EHEC among them during the training, since they participated in separate and different tasks. Three patients of Facility C are clustered on 7–8 June following the first case at the same facility on 1 June.

The predominant clinical symptoms and signs of the 14 symptomatic cases were diarrhoea (13/14 cases, 93%), including bloody diarrhoea (2, 14%); abdominal pain (6, 43%); nausea or vomiting (3, 22%); and fever ≥ 37.5 °C (1, 7%). Two patients (14%) developed HUS; however, both fully recovered. Of the 10 symptomatic patients (71%) who sought medical care, five (36%) were hospitalized. No residents or staff members died from this outbreak.

EHEC O157:H7 (VT2) was detected in the stool of 58 case patients. PFGE analysis found that all of the 28 randomly selected case patients had nearly identical strains with differences within two bands. (*11*) The strain was also later found in one patient in Nakatsu, about 100 km north-western of the institute in July 2005; however, an epidemiological investigation found no direct link between that case and cases detected in this outbreak.

All individuals with confirmed infection, including those who were asymptomatic, were treated with antibiotics and tested negative twice for EHEC O157:H7 48 hours after the end of treatment. One patient with HUS was treated with fosfomycin, and the other was treated with a fluoroquinolone before onset of HUS. Of 45 asymptomatic individuals, 37 took fosfomycin, six levofloxacin and one each tosufloxacin and cefpodoxime proxetil.

We studied potential risk factors for EHEC O157:H7 infection. Among the resident cases, 88% were independent with respect to feeding, and 54% were independent with respect to urinating. However, most residents required assistance with personal hygiene and grooming (assistance was required by 63% with hand washing, 67% with defecation, and 60% with brushing teeth). Some residents (16% of Facilities A and B) had problems with personal hygiene, in particular allotriophagic behaviour (i.e. eating one’s own feces) and manipulating their own feces.

Eleven kitchen staff members, including three nutritionists, worked in a single kitchen preparing meals for the residents and staff who ate in dining halls in each facility. Food items served to the residents and staff were almost identical. Three nutritionists took turns eating each meal, and none of them exhibited diarrhoea during the outbreak period. All the kitchen staff tested negative for EHEC in three separate stool samples collected on 20 May (just before the outbreak as part of routine screening for food-handlers), on 3–4 June and 8–9 June. Samples of the meals provided from 23 to 30 May were stored in a freezer, and all samples tested negative for EHEC. Water samples from the tap water were all culture negative for EHEC. Chlorine levels of the tap water were checked and recorded every day and were consistently greater than 0.1 ppm.

We investigated the pre-outbreak routines for cleaning the toileting areas. The toilets of Facilities A and B were cleaned every day by the staff, two toilets on the 1st floor of Facility C were cleaned every day by external vocational training participants and residents of Facility C, and toilets on the 2nd and 3rd floors of Facility C were cleaned by the residents. The cleaning was normally done with detergent applied with reusable mops and cloths. Gloves were not always used during the cleaning before the outbreak. The time spent cleaning each restroom was normally 20 to 30 minutes.

No animals were brought into the facilities before or during the outbreak period.

Upon the recognition of the outbreak, the facilities introduced additional disease prevention and control measures, including encouraging intensive hand washing before meals and after toilet use, increasing monitoring of residents with allotriophagic behaviour, strengthening daily diarrhoea surveillance and cleaning surfaces three times per day. Prior to the outbreak, an infection control protocol was developed; anecdotally, the protocol was not followed consistently.

The results of the primary univariable analyses are shown in **Table 2****,** and the stratified analyses for the residents of Facilities A and B are shown in **Table 3**. Additional univariable analyses are listed in **Table S1**. The univariable analysis found those residents who took meals at a certain table of Facility B were 9.7 (95% CI: 1.1–89.4) times the odds of being infected with EHEC (**Table S1B**). The inability to independently wash one’s own hands was significantly associated with being a case (OR: 5.3, 95% CI: 1.5–29.4), specifically in men (OR: 12.9, 95% CI: 1.8–562.6) and not women (OR: 1.5, 95% CI: 0.2–18.7) (**Table 3****)**. Those who were unable to independently wash their own hands were more likely to be cases regardless of allotriophagic behaviour (adjusted odds ratio [aOR]: 4.4, 95% CI: 1.2–16.0) or needing assistance in defecation (aOR: 4.3, 95% CI: 1.1–16.3). At Facility C, it was found that the residents who had cleaned the female restroom on the 1st floor had 18.0 (95% CI: 4.0–104.4) times the odds of being infected with EHEC than those who did not (**Table 2B**).

**Table 2:** Individual characteristics associated with EHEC O157:H7 infection in univariable analyses of an outbreak investigation at an institute for people with intellectual disabilities, Japan, 2005

Individual risk factor	Cases (%)	Controls (%)	OR	95%CI
**A. Residents of Facilities A and B (34 cases and 82 controls)**				
Female sex	8 (23.5)	22 (26.8)	0.8	0.3–2.3
Manipulates own faeces	7 (20.6)	16 (19.5)	1.1	0.3–3.1
Has habit of touching own buttocks	6 (17.6)	8 (9.8)	2.0	0.5–7.2
Has used a toilet outside the facility	6 (17.6)	5 (6.1)	3.3	0.8–14.7
Lies on the floor daily	6 (17.6)	10 (12.2)	1.7	0.4–5.6
Licks walls and tiles daily	3 (8.8)	1 (1.2)	7.7	0.6–415.4
Sucks one's fingers daily	7 (20.6)	15 (18.3)	1.2	0.4–3.4
**Engages in allotriophagic behaviour**	**10 (29.4)**	**9 (11.0)**	**3.3**	**1.1–10.5**
Loiters in the facility daily	7 (20.6)	17 (20.7)	1.0	0.3–2.9
Takes medication	14 (41.2)	30 (36.6)	1.2	0.5–3.0
**Being unable to wash own hands**	**31 (91.2)**	**54 (65.9)**	**5.3**	**1.5–29.0**
Being unable to take meals independently	4 (11.8)	21 (25.6)	1.3	0.1–1.3
Being unable to defecate independently	25 (73.5)	48 (58.5)	2.0	0.8–5.4
**Needs assistance or direction in defecating**	**16 (47.1)**	**21 (25.6)**	**2.6**	**1.0–6.4**
Needs assistance in bathing	20 (58.8)	46 (56.1)	1.0	0.4–2.4
In day care group A1*	4 (11.8)	10 (12.2)	1.0	0.2–3.7
In day care group A2*	6 (17.6)	7 (8.5)	2.3	0.6–8.7
In day care group B1*	5 (14.7)	6 (7.3)	2.2	0.5–9.3
In day care group B2*	3 (17.6)	7 (8.5)	1.0	1.0–4.9
In day care group S*	3 (17.6)	7 (8.5)	1.0	1.0–4.9
In day care group V*	5 (14.7)	5 (6.1)	2.6	0.6–12.2
B. Residents, participants and staff members in activities of Facility C (18 cases and 65 controls)**				
**Female sex**	11 (61.1)	19 (29.2)	3.7	1.1–13.3
Being a staff member	1 (5.6)	19 (30.2)	0.1	0.0–1.0
Toilet use location				
1st floor male restroom	6 (35.3)	28 (47.5)	0.6	0.2–2.1
**1st floor female restroom**	10 (58.8)	11 (18.6)	6.0	1.7–23.6
**2nd floor male restroom**	4 (23.5)	35 (59.3)	0.2	0.0–0.8
**2nd floor female restroom**	12 (70.6)	16 (27.1)	6.3	1.7–26.5
3rd floor male restroom	5 (29.4)	25 (42.4)	0.6	0.1–2.0
Toilet cleaned at				
1st floor male restroom	1 (5.9)	11 (18.6)	0.3	0.0–2.2
**1st floor female restroom**	10 (58.8)	4 (6.8)	18.0	4.0–104.4
Bathing				
At Facility C	10 (58.8)	43 (72.9)	0.5	0.2–2.0
Bathed in a bathtub	10 (58.8)	30 (50.8)	2.6	0.5–27.6
Frequently drank the hot water when bathing	1 (5.9)	6 (10.2)	0.4	0.0–4.1

CI = confidence interval, OR = odds ratio

* The day care groups A1 and A2 consisted of mainly residents of Facility A; B1 and B2 consisted mainly of those of Facility B; S consisted of those who were on occupational therapy; and V consisted of those who need more attention of the staff.

** Not all individuals provided responses, so the number of responses may not be the total numbers of cases and controls.

**Table 3:** Individual characteristics associated with EHEC O157:H7 infection among individuals not able to independently wash their hands during an outbreak among residents of Facilities A and B at an institute for people with intellectual disabilities, Japan, 2005 (34 cases and 82 controls)

**Stratified risk factors**	**Unable to wash hands independently**		**OR**	**95% CI**
	**No. (*n* = 85)**			
	**Cases (%)**	**Controls (%)**		
**Overall**	**31 (91.2)**	**54 (65.9)**	**5.3**	**1.5–29.4**
Sex				
**Male**	**25 (96.2)**	**40 (65.6)**	**12.9**	**1.8–562.6**
Female	6 (75.0)	14 (66.7)	1.5	0.2–18.7
**M-H adjusted**	**–**	**–**	**5.3**	**1.5–18.7**
Allotriophagic behaviour				
Yes	10 (100.0)	9 (100.0)	–	–
**No**	**21 (87.5)**	**45 (61.6)**	**4.3**	**1.1–24.6**
**M-H adjusted**	**–**	**–**	**4.4**	**1.2–16.0**
Needs assistance in defecation				
Yes	16 (100.0)	20 (95.2)	–	–
**No**	**15 (83.3)**	**34 (55.7)**	**3.9**	**1.0–23.2**
**M-H adjusted**	**–**	**–**	**4.3**	**1.1–16.3**

CI = confidence interval, OR = odds ratio, M-H = Mantel-Haenszel method

The risk factors with statistically significant odds ratios are emphasized with bold font.

The items with statistically significant odds ratios are emphasized with bold font.

## Discussion

We investigated an EHEC outbreak that occurred during 1–13 June 2005, affecting 59 residents and staff members of an institute for children and adults with intellectual disabilities. The source of the outbreak could not be identified; however, the infection may have spread through the environment contaminated with EHEC. The residents who cleaned a particular restroom at Facility C had 18 times the odds of testing positive for EHEC compared to those who did not, and neither samples from meal remnants nor stool samples from staff who worked in the kitchen yielded EHEC. At Facilities A and B, it is likely that the infection spread via person-to-person contact because those who were unable to wash their own hands were more at risk. Environmental contamination was also supported by the findings that no single peak in the epidemic curve was noted, no episodes were reported in which a possible single source of infection was suspected and limitations in personal and toilet hygiene were confirmed. EHEC spread through contaminated environments has been previously reported; (*7*) thus our findings are consistent with previous reports.

Infection spread via person-to-person contact is the leading cause of most EHEC outbreaks in institutional settings in Japan and elsewhere, particularly at day cares, schools (*12*-*15*) and homes for older people. (*16*) Foodborne infections (*17*) and infection spread through the environment were sometimes suspected but were not supported by analytic epidemiology. (*12*) Thus, this study is unique in that cleaning a certain restroom was implicated by analytical epidemiology as a possible common source.

In this outbreak, about two thirds of cases were asymptomatic. In Japan, active case-finding routinely includes testing asymptomatic contacts. (*13*, *18*) According to the national surveillance data, one third of EHEC cases in Japan were asymptomatic. (*19*) Over three fourths of the cases in our setting were adults, supporting a previous report that the proportion of cases that were symptomatic declined with age. (*13*) Additionally, the doses of EHEC bacilli were likely small and thus not everyone developed symptoms. Asymptomatic carriers or recovered patients may shed EHEC for more than 30 days; (*20*) however, humans are not considered as reservoirs. (*21*) During an outbreak in Australia in 2007, an asymptomatic sibling spread EHEC to another sibling who developed HUS. (*22*) The role of asymptomatic carriers of EHEC in outbreaks should not be underestimated.

Our study has both strengths and limitations. Since all the residents and staff members of the institution were tested for EHEC in their stools, we were able to identify infections that were asymptomatic. Although we believe most infections were transmitted through the environment at Facility C, the environmental specimens did not yield the pathogen, most probably because the environment, particularly the door knobs, floor and tables, were disinfected shortly before the environmental samples were collected for bacteriological tests. In addition, residents with intellectual disabilities may have limited ability to provide comprehensive behavioural or risk information, and thus recall and information biases are likely. To minimize these biases, we verified the participants’ responses with staff members’ records.

We recommend that in institutional settings, particularly those that accommodate people with intellectual disabilities, staff should pay close attention to personal and toilet hygiene of the residents, and restrooms should be cleaned as often as possible to reduce possible infection via contact with contaminated surfaces. The infective dose of EHEC is small (lower than 700 organisms). (*23*) Institutions should also have a symptomatic surveillance system and monitor trends in diarrhoea incidence among residents. Prefectural governments should strengthen their surveillance systems, including pathogen surveillance with routine PFGE tests, to detect potential outbreaks involving multiple prefectures. Local health offices should provide congregate settings, including health facilities, with training about communicable diseases to prevent outbreaks.
